# Niosomes of Ascorbic Acid and α-Tocopherol in the Cerebral Ischemia-Reperfusion Model in Male Rats

**DOI:** 10.1155/2014/816103

**Published:** 2014-08-28

**Authors:** Jaleh Varshosaz, Somayeh Taymouri, Abbas Pardakhty, Majid Asadi-Shekaari, Abodolreza Babaee

**Affiliations:** ^1^Department of Pharmaceutics, School of Pharmacy and Novel Drug Delivery Systems Research Centre, Isfahan University of Medical Sciences, P.O. Box 81745-359, Isfahan, Iran; ^2^Pharmaceutics Research Center, Neuropharmacology Institute, Kerman University of Medical Sciences, P.O. Box 76175-493, Kerman, Iran; ^3^Neuroscience Research Center, Neuropharmacology Institute, Kerman University of Medical Sciences, Kerman 7619813159, Iran; ^4^Anatomical Sciences Department, Afzali Pour Medical Faculty, Kerman University of Medical Sciences, P.O. Box 76175-493, Kerman, Iran

## Abstract

The objective of the present study was to prepare a stable* iv *injectable formulation of ascorbic acid and α-tocopherol in preventing the cerebral ischemia. Different niosomal formulations were prepared by Span and Tween mixed with cholesterol. The physicochemical characteristics of niosomal formulations were evaluated* in vitro*. For* in vivo *evaluation, the rats were made ischemic by middle cerebral artery occlusion model for 30 min and the selected formulation was used for determining its neuroprotective effect against cerebral ischemia. Neuronal damage was evaluated by optical microscopy and transmission electron microscopy. The encapsulation efficiency of ascorbic acid was increased to more than 84% by remote loading method. The cholesterol content of the niosomes, the hydrophilicity potential of the encapsulated compounds, and the preparation method of niosomes were the main factors affecting the mean volume diameter of the prepared vesicles. High physical stability of the niosomes prepared from Span 40 and Span 60 was demonstrated due to negligible size change of vesicles during 6 months storage at 4–8^°^C.* In vivo* studies showed that ST60/Chol 35 : 35 : 30 niosomes had more neuroprotective effects against cerebral ischemic injuries in male rats than free ascorbic acid.

## 1. Introduction

Cerebral ischemia is the third leading cause of deaths in the United States [1]. In patient with acute cerebral ischemia, thrombolysis therapy could reduce death after ischemia [2]. Recombinant tissue plasminogen activator (r-TPA) is the only FDA approved drug which may be effective in 3 hours of symptom onset [3]. However, intracerebral hemorrhage (ICH) and mortality risk with r-TPA have led to development of alternative therapies [2]. Brain is a susceptible tissue to oxidative stress due to high level of free radicals, high amounts of unsaturated fatty acids, and fair protective antioxidant capacity in different parts [4]. It is reported that free radical oxygen has potential role in neural cell damage in ischemia-reperfusion disorders [5, 6]. Exogenous antioxidant such as ascorbic acid (vitamin C), α-tocopherol (vitamin E), and *β*-carotene can be effective on neuronal cell protection due to the effect of reactive oxygen species (ROS) on neuronal cell damages and fast consumption of endogenous scavenging antioxidants [5, 7]. But their effectiveness depends on their ability for transporting through the blood brain barrier (BBB) [8]. It was reported that oral administration of α-tocopherol supplementation despite making high plasma level could not increase α-tocopherol level in ventricular cerebrospinal fluid even at high doses which could be related to limited passage from BBB [9]. On other hand, ascorbic acid (a hydrophilic molecule) cannot transport efficiently across the BBB due to its polarity and hydrophilicity [8]. To overcome these problems, different drug delivery systems such as dendrimers [10], liposomes [11], poly-butyl cyanoacrylate (PBCA) nanoparticles coated with polysorbate 80 [12], human serum albumin [13], solid lipid nanoparticles [14], and polymeric nanoparticles such as poly(lactide-co-glycolic acid) ones [15] have been studied for increasing transport of different therapeutics across BBB. Liposomes containing ascorbic acid or α-tocopherol were prepared by Sinha et al. [5]. The results were encouraging, but low chemical stability and thermo-liability of phospholipids, the main constituents of liposomes, made us develop niosomes of these two supplements which are more stable to chemicals and temperature compared to liposomes.

Noisomes are nonionic surfactant vesicles used as drug carrier similar to liposomes. The low cost, greater stability, and resultant ease of storage of nonionic surfactants have led to the development of these carriers as alternatives to liposomes [16]. There are some reports about the increased level of niosomal encapsulated drugs in brain of animals [17]. Increased uptake of methotrexate was observed after intravenous administration of methotrexate niosomal formulation in mice [18]. Dufes et al. [19] successfully used glucose-bearing niosomes as a brain targeted delivery system for the vasoactive intestinal peptide (VIP). It was also reported that niosomal formulation of doxorubicin functionalized with the glucose-derivative N-palmitoyl glucosamine was able to improve doxorubicin brain concentration in contrast to its commercial solution [20]. The successful ability of niosomal carriers to transport through BBB and the capability of niosomes in encapsulation of both hydrophilic and lipophilic compounds provided our rational for designing of niosomal formulation of antioxidant of vitamin C and E for brain delivery in preventing stroke. This carrier also provides the ability of* iv* administration of lipid soluble molecules such as vitamin E.

Considering the more physical stability of niosomes than liposomes and regarding promising results of cerebral ischemia preventive effects reported for ascorbic acid and vitamin E liposomes [5], the main goal of our study was to prepare niosomal formulation of α-tocopherol and ascorbic acid for enhanced brain delivery of these drugs in preventing neuronal cell damages during ischemia-reperfusion disorders. Different pharmaceutical parameters such as particle size, encapsulation efficiency,* in vitro* release of encapsulated materials, and stability of the designed formulations were evaluated. In addition, the neuroprotective effects of the designed formulations were investigated in an ischemia-reperfusion model in male rats. To our knowledge, there is no report on the production and application of niosomal formulation of these two vitamins in prevention of ischemia-reperfusion model in rats.

## 2. Materials and Methods

### 2.1. Materials

Ascorbic acid and α-tocopherol were purchased from Merck Chemical Company (Germany). The nonionic surfactants used as vesicle-forming materials including polysorbate 40 (Tween 40), polysorbate 60 (Tween 60), sorbitan monopalmitate 40 (Span 40), sorbitan monostearate 60 (Span 60), and cholesterol (Chol) were purchased from Fluka Company (Switzerland). All organic solvents and the other chemicals were of analytical grade and obtained from Merck Chemical Company (Germany).

### 2.2. Preparation of Drugs-Loaded Niosomes

#### 2.2.1. Conventional Film Hydration Method

Vesicular formulations containing ascorbic acid or α-tocopherol were prepared by film hydration method [21]. The composition of different niosomal formulations is shown in Table 1. Briefly, niosomes of ascorbic acid were prepared by dissolving 400 *μ*mol of surfactants (equal molar percent of Tween/Span with the same hydrocarbon chain type and length)/Chol in chloroform in a round-bottomed flask. The organic solvent was evaporated under reduced pressure at 55°C. The resultant thin lipid film produced on the inner wall of the flask was then hydrated with 10 mL of normal saline 0.9 w/v% solution containing 2.5 mg/mL of ascorbic acid at 55°C for 30 min. α-Tocopherol niosomal formulations were prepared by the same amounts and types of surfactants/Chol and the same method as ascorbic acid but 20 mg of α-tocopherol was dissolved in the chloroform along with the other lipids in the round-bottomed flask. The compositions of the different formulations of vesicles are listed in Table 1.

#### 2.2.2. Remote Loading Method

Ascorbic acid was loaded into preformed niosomes by modifying a remote-loading technique [22–24]. The composition of different niosomal formulations is shown in Table 1. Briefly, 400 *μ*mol of surfactants and cholesterol was dissolved in chloroform in a round-bottomed flask. The organic solvent was evaporated under reduced pressure at 55°C. The dried lipid film was hydrated with 5 mL calcium acetate (200 mM). Nonentrapped calcium acetate was removed from the niosomal suspension by dialysis (molecular weight cutoff of 12 kDa) against dextrose 5 w/v% for 2 h. Then, 5 mL of the solution of ascorbic acid with concentration of 5 mg/mL was added to the prepared niosomal suspension and mixed in a rotating water bath for 20 min.

### 2.3. Vesicle Size Measurement

The particle size of different formulations was measured using a static laser light diffraction method by Malvern particle size analyzer (Malvern Instruments, MasterSizer 2000E, UK) 48 h after preparation.

### 2.4. Encapsulation Efficiency Determination

To separate nonentrapped α-tocopherol or ascorbic acid from niosomes, the vesicular suspensions were centrifuged (National labnet, USA) at 25000 rpm for 60 min at 4°C and washed twice with normal saline 0.9 w/v%. The amount of entrapped α-tocopherol or ascorbic acid in the niosomes was analyzed by UV/visible spectrophotometer (Shimadzu 2100, Japan) at *λ*_max⁡_ of 246 and 290 nm for ascorbic acid and α-tocopherol, respectively, after disrupting the niosomes by ethanol 96%. The encapsulation efficiency percent of α-tocopherol or ascorbic acid (EE %) was determined from

(1) $$\begin{array}{c} \text{EE}\%\;=\;\left(\frac{C_{p}}{C_{T}}\right)\;\times \;100, \end{array}$$

where *C*_*p*_ is the active component concentration encapsulated in the niosomes and *C*_*T*_ is the initial drug concentration added to formulation. Empty niosomes were used as blank.

### 2.5. *In Vitro* Drug Release Studies

Ascorbic acid release from various formulations was evaluated using dialysis method. The dialysis membrane (molecular weight cutoff of 12 kDa) that contained 4 mL of ascorbic acid formulation was placed in a glass flask filled with 40 mL of normal saline 0.9 w/v% as receptor compartment. Temperature was maintained at 37 ± 1°C by a circulating water bath. The medium in the receptor compartment was magnetically stirred at a rate of 100 rpm. Samples of the medium were withdrawn at fixed time intervals and replaced with an equal volume of fresh normal saline for 6 h. The drug released concentration in the medium was quantified spectrophotometrically. In the case of lipid soluble material, α-tocopherol, drug release was negligible in the mentioned period of release test due to the low partitioning characteristics of vitamin E in aqueous media and intercalating of this material in lipid bilayers.

### 2.6. Physical Stability of Vesicles

The vesicles were stored in glass vials in refrigerator (4–8°C) for 6 months and the changes in vesicles diameter were determined by laser light scattering method. The changes in morphology of multilayered vesicles (MLVs) and also the separation of constituents were assessed by the optical microscope (HFX-DX, Nikon, Japan). No special precautions were taken to improve the stability of the vesicles.

### 2.7. *In Vivo* Studies

#### 2.7.1. Pretreatment of Animals with Niosomes and Induction of Cerebral Ischemia

Male Wistar rats weighing 220–270 g were used for study. Animals had free access to food and water before and after surgical procedures. All animal studies were done in compliance with the ethics guidelines approved by the Kerman University of Medical Science (Kerman, Iran). Rats were divided into 7 groups: group 1 for ascorbic acid niosomal formulation, group 2 for α-tocopherol niosomal formulation, group 3 for free ascorbic acid (as α-tocopherol was not water soluble there was no treated group with* iv* administration of free α-tocopherol), group 4 for normal saline (negative control), group 5 for blank niosomes, group 6 for a mixture of α-tocopherol and ascorbic acid niosomes in a ratio of 1 : 1 (w/w), and group 7 for sham-operated rats. All animals received either ascorbic acid or α-tocopherol with a dose of 8 mg/kg body weight which was injected into the tail vein of rats 2-3 h before cerebral ischemia [5]. Animals were anesthetized with chloral hydrate (400 mg/kg) and were made ischemic by middle cerebral artery occlusion (MCAO) for 30 min [25]. Under the neurosurgical microscope, the right common carotid artery (CCA) was exposed to a midline incision. After blocking all branches of the external carotid artery (ECA) and extra cranial branches of the internal carotid artery (ICA), a 4–0 nylon intraluminal suture was introduced into cervical ICA and advancing in intracranially to block blood flow into the middle cerebral artery (MCA). Following thirty minutes, suture was withdrawn and blood flow resumed. After recovery from the anesthesia, the animals were returned to their home cages. Two days after ischemia, the animals were tested for neurological examination. Neuronal damage was evaluated by optical microscopy and was estimated as a rate of the number of degenerated pyramidal neurons to that of both surviving and degenerated in three distinct areas of the cortex subfield in coronal sections for each animal [26]. The morphology of neurons was evaluated under transmission electron microscope (EM300, Philips, Holland).

## 3. Results and Discussion

### 3.1. Encapsulation Efficiency

Encapsulation efficiencies (EE) in all studied formulations are shown in Table 2. α-Tocopherol had often higher EE% than ascorbic acid, which is clearly due to its hydrophobic nature and its intercalating in lipophilic core of surfactant bilayers [27]. Increasing the amount of Chol from 30 to 50 mole percent reduced the EE% of α-tocopherol in both types of studied surfactants which was due to the competition between α-tocopherol and Chol molecules. Remote-loading procedure by using an ammonium sulfate gradient method [28] and complexation with arylsulfonates are two ways reported for intravesicular precipitate and enhancement of drug retention [29]. In our study, higher encapsulation efficiency of ascorbic acid was achieved by the remote-loading procedure by using calcium acetate gradient method (Table 2) compared to the other loading method (*P* < 0.05). It was supposed that during remote loading ascorbic acid diffused in uncharged form via niosome membrane due to calcium acetate and pH trans-membrane gradient. There, it loses its proton, becomes negatively charged, and forms a poorly soluble ascorbate calcium salt, which precipitates in the intraniosomal aqueous phase. This method also improved encapsulation efficiency of ciprofloxacin [30], diclofenac, insulin, and fluorescein isothiocyanate labeled insulin [22] in liposomes and luteinizing hormone releasing hormone niosomes [31]. Increasing the molar ratio of Chol from 30 to 40 increased the EE% of ascorbic acid significantly (*P* < 0.05) (Table 2). This can be related to increasing the rigidity of the bilayers following Chol content enhancement which is parallel to gel transition temperature abolishment following Chol incorporation in lipid bilayers [32].

### 3.2. Morphology and Size Distribution of Vesicles

**Figure 1:**
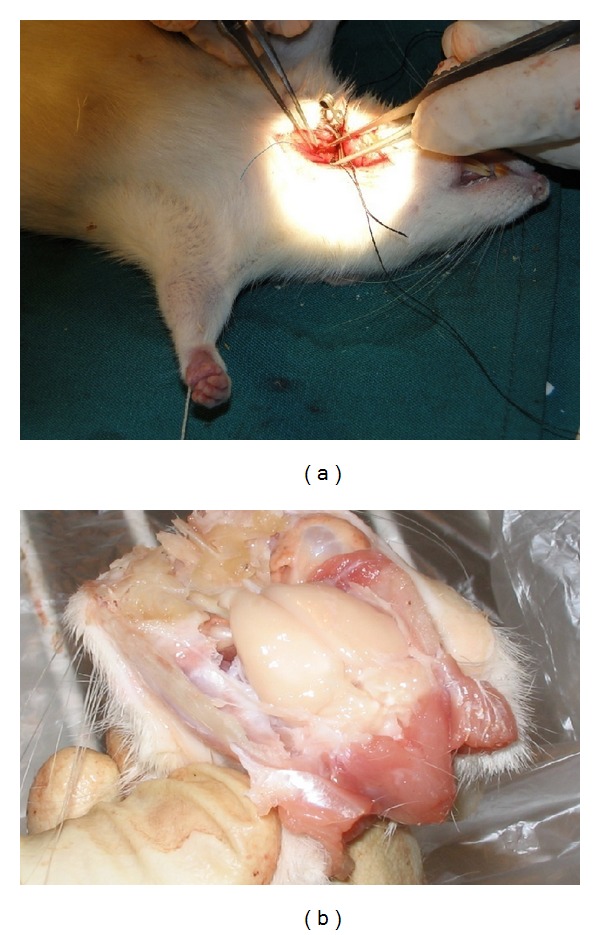
Surgical technique of middle cerebral artery occlusion in rat.

**Figure 2:**
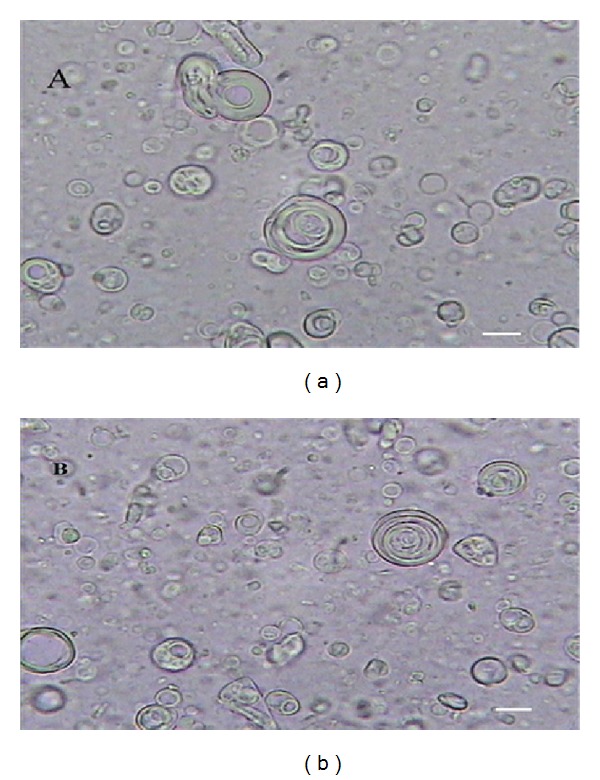
Optical micrographs of ascorbic acid or α-tocopherol containing niosomes (×400 magnification). Niosomes were composed of (a) Span Tween 40/cholesterol (30 : 30 : 40 mole%) ascorbic acid, (b) Span Tween 60/cholesterol (35 : 35 : 30 mole%) α-tocopherol. (Scale bar: 5 *μ*m).

All used nonionic surfactant compositions formed niosomes in the presence of cholesterol.  Figure 2 demonstrates the formation of niosomes in different formulations. Morphologically, the formulated niosomes were frequently as round MLVs as depicted in Figure 1. Obviously, this was predictable as film hydration method usually produces MLVs.

The mean volume diameters (*d*_*v*_) of the prepared nonionic surfactant vesicles with different compositions are presented in Tables 3 and 4. From data presented in these tables, it is obvious that, following the increase of cholesterol molar percent, the *d*_*v*_ of neutral niosomes prepared by film hydration or remote loading methods was increased. Moazeni et al. [33] also reported the forming of the greater ciprofloxacin niosomes after raising the cholesterol content from 30 to 40 and 50 molar percent. The integration of the drug also has a significant effect on the particle size of the vesicles. It was revealed that the incorporation of vitamin C or vitamin E in all formulations led to size reduction compared to empty niosomes (*P* < 0.05) (the results of empty vesicles were not shown here). In a similar manner, Junyaprasert et al. [34] reporeted a slight size reduction in niosomal suspensions composed of Span 60/Chol in citrate buffer (pH 5) following the encapsulation of salicylic acid. Another important factor that affected the particle size of vesicles was the method of preparation. The mentioned niosomes prepared by film hydration technique were significantly smaller than remote loading vesicles (*P* < 0.05). In ascorbic acid, niosomal formulations prepared from Span/Tween 40 and at the low levels of Chol (30 and 40 molar ratios), the vesicles volume diameter was more influenced by the preparation method as depicted in Table 3. The difference in dimensions of Span/Tween 60 niosomes prepared by two methods is also seen in Table 2 but with less extent. More rigidity of the bilayers composed of stearyl alkyl chain (*C*_18_) in Span/Tween 60 niosomes in comparison to palmityl alkyl chain (*C*_16_) of Span/Tween 40 may explain this finding.

### 3.3. *In Vitro* Release Studies

**Figure 3:**
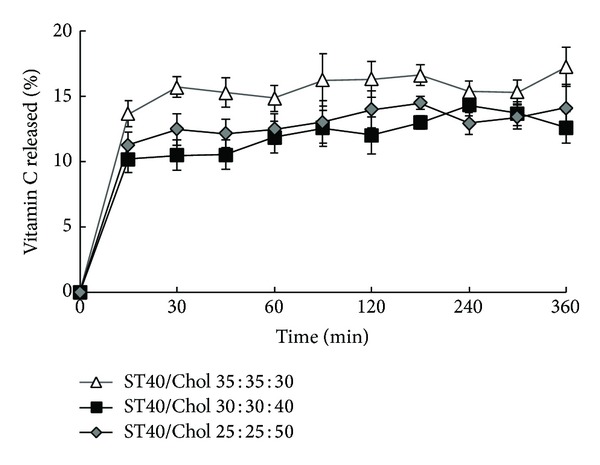
Release profiles of ascorbic acid from niosomes composed of Span-Tween 40/Cholesterol in normal saline at 37°C (mean ± SD, *n* = 3).

**Figure 4:**
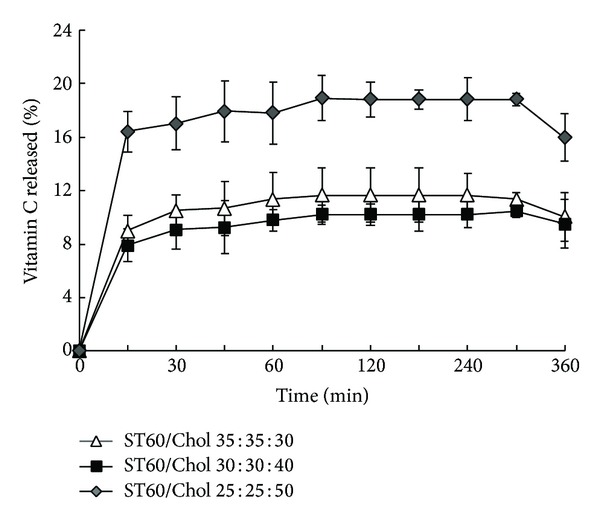
Release profiles of ascorbic acid from niosomes composed of Span-Tween 60/Cholesterol in normal saline at 37°C (mean ± SD, *n* = 3).

The type of entrapped molecule, vesicle lamellarity, and presence or absence of the charging agents must be considered in drug permeation studies in vesicular systems. The chemical structure of bilayer forming lipids has clear effect on drug efflux from vesicles as well. In many cases, the drug release profile from niosomal systems are biphasic such as caffeine [35] and insulin [32] indicating a rapid desorption and a slower diffusion of entrapped drug though bilayer phases. In the present study, similar biphasic release was observed (Figures 3 and 4). The rapid initial phase may be related to desorption of drug from the surface of niosomes. After the initial burst release, a constant ascorbic acid release was observed during 360 min which was due to diffusion of ascorbic acid from lipid bilayer. According to this result, it took time for ascorbate ion to be released from multiple bilayers of niosomal vesicles that were stabilized by cholesterol. Interestingly, the overall release amount of ascorbic acid is adjustable with EE% of drug in which more EE% led to less drug release.

### 3.4. Stability Studies

**Figure 5:**
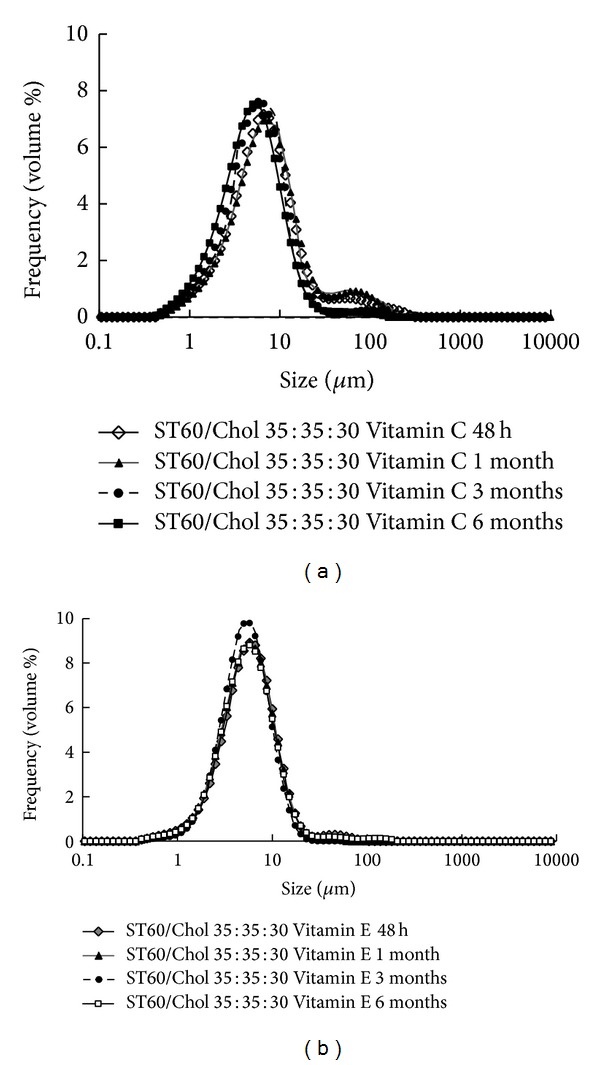
The size distribution changes of niosomes composed of (a) Span-Tween 60/Cholesterol 35 : 35 : 30 contining vitamin C, (b) Span-Tween 60/Cholesterol 35 : 35 : 30 contining vitamin E during storage at 4°C as an indicator of physical stability of vesicles.

Tables 3 and 4 exhibit the change in particle size is as a major indicator for niosomes stability during storage at 4°C for 6 months. In the present study, high vesicular stability was observed as depicted in Figure 5. Higher stability in vesicular structure of formulations with more cholestrol content was seen (*P* < 0.05) (Tables 2 and 3). The role of cholestrol in increasing membrane stability was reported perviously [33]. On the other hand, relatively large diameter of all prepared niosomes (>5 *μ*m) maybe another reason for observed vesicular stability during 6-month storage at refrigerator temperature because smaller vesicles are thermodynamically unstable [33]. The last reason of our niosomal stability could be related to gel state nature of Span/Tween 40 and Span/Tween 60 bilayers which are less sensitive to temperature fluctuation and less desire for bilayer fusion. In some cases such as Span/Tween 40/Chol/vitamin E (50 molar ratio of Chol), a slight decrease in mean volume diameter of vesicles was observed (Tables 3 and 4). Similar results were observed in formulations prepared from Brij 52 and cholesterol [32]. To explain this observation, two theories are suggested [36]: (i) the ions adsorption to the bilayer originates a change in the head groups charge and, as a consequence, the bilayer changes its curvature due to the electric interactions which in turn causes changing in their size and (ii) the concentration gradient at both membrane sides generates an osmotic force due to the membrane impermeability to some ions, the vesicles react sending off water, decreasing their size.

### 3.5. Animal Studies

**Figure 6:**
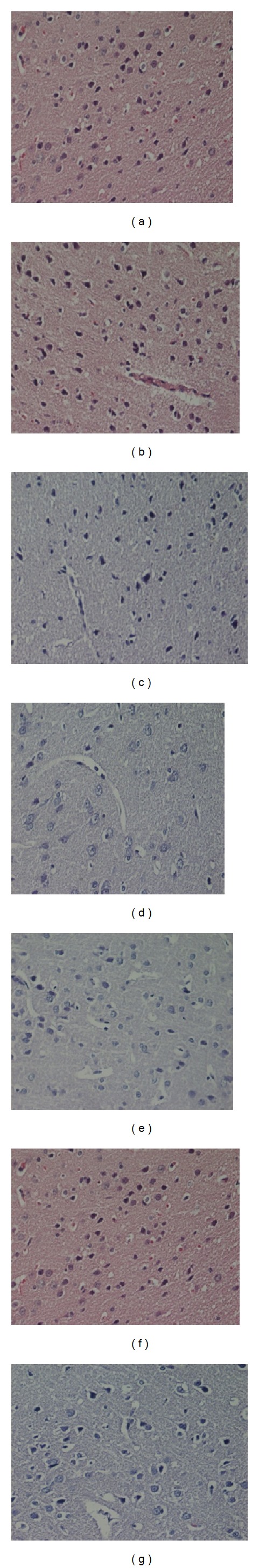
Histological changes in brain cortex from (a) sham-operated, (b) empty noisome, (c) saline-treated, (d) free ascorbic acid, (e) ascorbic noisome, (f) α-tocopherol noisome, and (g) niosome formulation with a mixture of α-tocopherol and ascorbic acid in a ratio of 1 : 1 (w/w) treated rats. As shown in the figure, morphology of the neurons in sham-operated group is intact. Severe degenerative changes (dark nucleus and shrunken cytoplasm) are present in the saline-treated and empty noisome groups. These changes were less in the other groups, particularly in the niosomal formulation with a mixture of α-tocopherol and ascorbic acid. (Magnification ×400).

According to the obtained data in light microscopy study (Figure 6), the morphology of neurons in sham-operated group was normal but the most of the pyramidal neurons in saline-treated and blank niosome groups showed severe degenerative changes including eosinophilic and shrunk cytoplasm with extensively dark picnotic nuclei. In experimental groups that received the drug, the severity of degenerative changes in cytoplasm and nucleus were significantly less compared to saline-treated groups and groups that received blank niosome (*P* < 0.05). In addition, encapsulated niosomal formulations especially the mixture of α-tocopherol and ascorbic acid niosomal formulation (1 : 1 w/w 4 mg/kg) showed more positive effects in cerebral-induced injuries (Figure 6). In experimental groups receiving ascorbic acid, loaded niosome and a combination of ascorbic acid and α-tocopherol niosomal formulation, the mean number of degenerated neurons were significantly smaller than free ascorbic acid (*P* < 0.05). However, the difference between the two niosomal groups were not statistically significant (*P* > 0.05).

For confirmation of our results in light microscopy, we also studied the ultrastructure of the pyramidal neurons in the cortex of male rats using transmission electron microscope (TEM).

**Figure 7:**
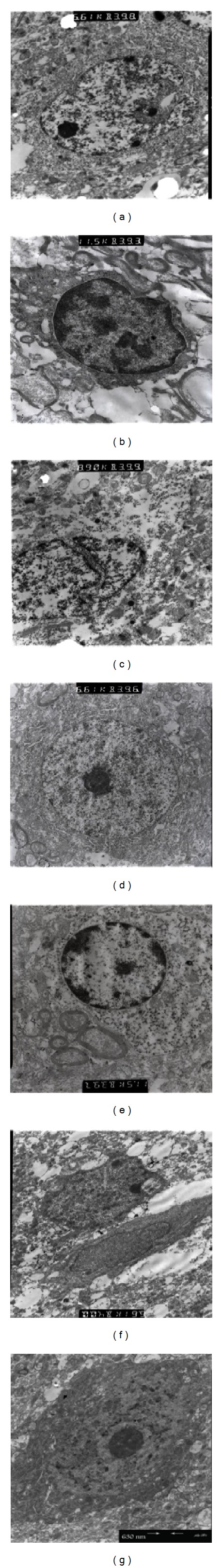
Electron micrograph of cortical neurons from (a) sham-operated, (b) empty noisome, (c) saline treated, (d) free ascorbic acid, (e) ascorbic noisome, (f) α-tocopherol noisome, and (g) niosomal formulation with a mixture of α-tocopherol and ascorbic acid treated rats. As shown in the micrograph, the normal ultrastructure of cortical neuron is visible in sham-operated group. Ischemia-reperfusion resulted in severe degenerative changes including chromatin aggregation, organelles swelling of the neurons in the saline-treated and empty niosome groups. Meanwhile, the whole ultrastructure of cortical neurons was maintained in the other groups mostly in the niosome formulation with a mixture of α-tocopherol and ascorbic acid. (Scale bar: 630 nm).

Morphology of cortical neurons in sham-operated group was intact (Figure 7). Some degenerative changes including organelles swelling, chromatin aggregation, and darkening of nucleus were observed in saline-treated and blank noisome groups. Treatment with prepared drugs preserved the ultrastructure of the most cortical neurons especially the mixture of α-tocopherol and ascorbic acid niosomal formulation (1 : 1 w/w 4 mg/kg) (Figure 7). In other words, the electron microscopy results confirmed the data obtained by light microscopy.

## 4. Conclusions

To compensate the problems related to transport of natural antioxidants in brain ischemia due to stroke, two different compounds of α-tocopherol and ascorbic acid were formulated in niosomes composed of sorbitane esters and their ethoxylated derivatives. The cholesterol content and the hydrophilicity potential of encapsulated compounds were the main factors affecting the mean volume diameter of the prepared vesicles. Furthermore, in ascorbic acid niosomes, the method of niosome preparation had obvious effect on the mean diameter of vesicles. High physical stability of gel state niosomes caused negligible size change during 6-month storage at 4–8°C.

*In vivo* results showed that although there was no significant difference between the mean number of degenerated neurons in the group received ascorbic acid niosomes with those treated with a mixture of ascorbic acid and α-tocopherol niosomes and the groups treated with α-tocopherol niosomes alone (*P* > 0.05) but, it was significantly higher in the group receiving free ascorbic compared to the mixed niosomes. In other words, the effectiveness of the formulated new drug delivery system in protection of cerebral tissue against elevation in oxygen free radical concentration during cerebral ischemia-reperfusion course was more than the free ascorbic acid. In addition, the designed niosomal formulation provides a suitable possible way for *iv* administration of a water insoluble drug like α-tocopherol.

## Figures and Tables

**Table 1:** Composition of different niosomal formulation containing ascorbic acid or *α*-tocopherol.

Formulation code	Cholesterol	Span	Tween	Span	Tween
Molar ratio		40	40	60	60
ST40/Chol 35 : 35 : 30	30	35	35	—	—
ST40/Chol 30 : 30 : 40	40	30	30	—	—
ST40/Chol 25 : 25 : 50	50	25	25	—	—
ST60/Chol 35 : 35 : 30	30	—	—	35	35
ST60/Chol 30 : 30 : 40	40	—	—	30	30
ST60/Chol 25 : 25 : 50	50	—	—	25	25

**Table 2:** Encapsulation efficiency of different formulations containing vitamin C and vitamin E.

Niosomal formulations	Vitamin C encapsulation efficiency %		Vitamin E encapsulation efficiency %
	Remote loading	Film hydration	Film hydration
ST40/Chol 35 : 35 : 30	55.24 ± 0.17	7 ± 3.56	88.136 ± 4.90
ST40/Chol 30 : 30 : 40	70.54 ± 0.14	7.66 ± 1.2	83.51 ± 7.60
ST40/Chol 25 : 25 : 50	66.68 ± 2.47	8.53 ± 0.12	65.30 ± 12.13
ST60/Chol 35 : 35 : 30	79.63 ± 0.56	10 ± 0.634	91.5 ± 3.54
ST60/Chol 30 : 30 : 40	85.94 ± 5.47	9.11 ± 1.54	82.17 ± 18.63
ST60/Chol 25 : 25 : 50	48.36 ± 9.41	9.63 ± 2.34	56.85 ± 9.27

**Table 3:** Mean volume diameter of ascorbic acid loaded niosomes prepared by film hydration method after 48 h and remote loading method at different time intervals after preparation and storage at 4°C (mean ± SD, *n* = 3).

Niosomal formulations	Film hydration *d*_*v*_ (*μ*m) ± SD		Remote loading *d*_*v*_ (*μ*m) ± SD		
	48 h	48 h	1 month	2 months	6 months
ST40/Chol 35 : 35 : 30	5.06 ± 0.13	9.10 ± 0.84	7.80 ± 0.11	7.83 ± 0.429	6.87 ± 0.106
ST40/Chol 30 : 30 : 40	7.55 ± 0.38	9.14 ± 0.25	8.25 ± 0.15	8.00 ± 0.21	7.921 ± 0.354
ST40/Chol 25 : 25 : 50	8.73 ± 0.05	8.87 ± 0.13	8.48 ± 0.08	8.38 ± 0.130	8.03 ± 0.159
ST60/Chol 35 : 35 : 30	6.95 ± 0.07	7.06 ± 0.13	7.15 ± 0.64	6.03 ± 0.098	5.225 ± 0.057
ST60/Chol 30 : 30 : 40	9.35 ± 0.59	8.78 ± 0.29	7.87 ± 0.13	7.49 ± 0.50	7.069 ± 0.168
ST60/Chol 25 : 25 : 50	9.04 ± 0.34	9.06 ± 0.14	9.48 ± 0.81	8.98 ± 0.054	8.717 ± 0.101

**Table 4:** Mean volume diameter of *α*-tocopherol loaded niosomes at different time intervals after preparation and storage at 4°C (mean ± SD, *n* = 3).

Niosomal formulations	*d*_*v*_ (*μ*m) ± SD			
	48 h	1 month	2 months	6 months
ST40/Chol 35 : 35 : 30	7.60 ± 0.40	8.11 ± 0.38	7.15 ± 0.32	8.62 ± 0.32
ST40/Chol 30 : 30 : 40	8.14 ± 0.36	8.07 ± 0.29	7.23 ± 0.25	9.17 ± 0.22
ST40/Chol 25 : 25 : 50	9.86 ± 0.12	9.61 ± 0.08	9.22 ± 0.14	9.20 ± 0.19
ST60/Chol 35 : 35 : 30	6.42 ± 0.08	6.02 ± 0.17	5.85 ± 0.27	6.11 ± 0.24
ST60/Chol 30 : 30 : 40	9.36 ± 0.47	7.19 ± 0.29	8.48 ± 0.49	10.22 ± 0.27
ST60/Chol 25 : 25 : 50	7.67 ± 0.07	8.47 ± 0.09	7.47 ± 0.1	7.42 ± 0.10
